# Prevalence of HCV and/or HBV coinfection in Iranian HIV-infected patients

**DOI:** 10.2217/fvl-2019-0066

**Published:** 2020-04-24

**Authors:** Farzaneh Dehghani-Dehej, Zinat Hosseini, Poupak Mortazkar, Khadijeh Khanaliha, Maryam Esghaei, Atousa Fakhim, Farah Bokharaei-Salim

**Affiliations:** 1 ^1^Department of Virology, School of Medicine, Iran University of Medical Sciences, Tehran, Iran; 2 ^2^School of Medicine, Iran University of Medical Sciences, Tehran, Iran; 3 ^3^Research Center of Pediatric Infectious Diseases, Institute of Immunology & Infectious Diseases, Iran University of Medical Sciences, Tehran, Iran; 4 ^4^Department of Architectural Engineering, Faculty of Engineering, Islamic Azad University, South Tehran Branch, Tehran, Iran; 5 ^5^Master of Science of Virology, Iran University of Medical Sciences, Tehran, Iran; 6 ^6^General Medical Student, School of Medicine, Iran University of Medical Sciences, Tehran, Iran; 7 ^7^PhD Student of Virology, Department of Virology, School of Medicine, Iran University of Medical Sciences, Tehran, Iran; 8 ^8^Assistant Professor of Parasitology, Research Center of Pediatric Infectious Diseases, Institute of Immunology & Infectious Diseases, Iran University of Medical Sciences, Tehran, Iran; 9 ^9^Associate Professor of Virology, Iran University of Medical Sciences, Tehran, Iran; 10 ^10^Student of Architectural Engineering, Islamic Azad University, South Tehran Branch, Tehran, Iran; 11 ^11^Assistant Professor of Virology, Iran University of Medical Sciences, Tehran, Iran

**Keywords:** coinfection, HBV, HCV, HIV

## Abstract

**Aim:** HIV-infected patients risk coinfection with HBV and HCV. This study aimed to investigate molecular epidemiology of HBV and HCV coinfection in Iranian HIV-infected individuals. **Materials & methods:** In this cross-sectional study, serological markers of HBV and HCV infection (hepatitis B surface antigen [HBsAg], hepatitis B e-antigen [HBeAg], hepatitis B e-antibody [HBeAb] and hepatitis B core antibody [HBcAb]) and anti-HCV antibodies [anti-HCV Abs] were tested in 198 Iranian HIV-infected patients. From plasma, HBV viral load was determined using COBAS TaqMan 48, and HCV-RNA was detected by reverse transcriptase-nested PCR. **Results:** 85 out of 198 (42.9%) patients were anti-HCV Ab positive and 42/198 (21.2%) had detectable HCV-RNA. Eight (4.0%) had traceable HBV-DNA. All these patients were infected by HBV genotype D. 55 (27.8%) were HBcAb positive. Nine (4.4%) were HBsAg and anti-HCV Ab positive. **Conclusion:** None were HIV-RNA/HCV-RNA/HBV-DNA positive, 21.2% were HIV-RNA/HCV-RNA positive and 4.0% were HIV-RNA/HBV-DNA positive. Therefore, studies on diagnosing these infections in HIV-infected individuals may be valuable.

The HIV is a member of the *Retroviridae* family and *Lentivirous* genus [1]. More than 37 million people worldwide are living with HIV infection [2]. HIV-infected individuals are at risk of other blood-borne viruses such as HBV and HCV. Each of these infections is a major problem for the WHO [3].

HBV is a virus belonging to *Hepadnaviridae* family and *Orthohepadnavirus* genus. This virus is partially dsDNA containing a circular genome about 3.2 kb in size, and encodes seven viral proteins [4]. There are four open-reading frames in the genome (C, X, P and S) [5,6]. 10 genotypes of HBV (A–J) are currently recognized. Structural differences between genotypes can affect the response to treatment of HBV infection and presumably vaccination against the virus [7]. More than 250 million people with chronic HBV infection are at risk of developing liver disease [8]. The estimated prevalence of HBV infection in Iran was about 2.2% (95% CI: 1.9–2.6%) [9].

HCV is a single-stranded, positive-sense RNA virus, approximately 9.6 kb in length. This virus is a member of the *Flaviviridae* family and *Hepacivirus* genus [10]. To date, eight genotypes and a large number of subtypes of HCV are recognized [11]. Differences between genotypes can affect the response to treatment and treatment duration of HCV infection [12]. HCV genotyping has public health significance as it can be useful for checking outbreaks and epidemiology of the infection [11,12]. Hepatitis C has a global impact in terms of mortality and morbidity with over 70 million people infected all around the world [13]. According to studies in Iran, the prevalence of HCV infection is nearly 0.5% (1.0% in men and 0.1% in women) [14,15]. For HCV infection and the liver damage associated with it, the leading cause of mortality and morbidity is among HIV-infected patients. According to available evidence, HIV/HCV-coinfected patients are at higher risk for liver cirrhosis and hepatocellular carcinoma (HCC) [16,17].

Given that the transmission routes of HIV, HBV and HCV viruses are common, these infections can occur simultaneously. Worldwide, nearly 40 million people are living with HIV, about 2.6 million people are infected with HBV and about 2.8 million people are HCV-infected [2].

HIV infection intensifies natural history of HBV infection, which can lead to an increase in rates of HBV persistence, relapse of HBV (resurgence of hepatitis B surface antigen [HBsAg], hepatitis B e-antigen [HBeAg] or HBV-DNA) and considerable clinical disease. Previous studies of the HBV/HIV coinfection have shown that HIV leads to a lack of protective immunity against HBV, increased risk of cirrhosis and HCC and liver-related mortality [18,19].

The effect of antiretroviral therapies (ARTs) on the natural history of HBV-related disease have been different, in some studies, it leads to recovery from HBV infection and in other studies, with relapse of hepatitis B [18,20].

The death rate in HIV-positive patients decreased after taking combination ARTs, but only in those with HIV/HBV or HIV/HCV coinfection. The mortality rate is high due to liver damage. HIV/HBV-coinfected people have a higher rate of progression to liver fibrosis, cirrhosis, HCC, less clearance of HBsAg and occult HBV infections (OBI) are more frequent in these patients [13].

Therefore, it seems that screening for HBV infection in the HIV-infected individuals should be done. Testing HBsAg, HBeAg and Ab and determining HBV viral load are an essential part of HBV infection assessment in HIV/HBV-coinfected patients. Obviously, determination of CD4 counts and HIV viral load are necessary along with the antiretroviral drug-resistant response [21].

According to evidence, HCV/HIV-coinfected patients are at higher risk for cirrhosis and HCC [22]. HIV infection exacerbates natural history of HCV infection. HCV-RNA loads in these patients are higher and clearance of hepatitis C viremia after acute infection in HIV-positive patients are less, and liver diseases in these patients show more progress than patients with HIV infection alone [20].

It is known that HBV and HCV infections have been associated with various clinical manifestations in people with HIV infection including impaired immune response during ARTs, and also increased susceptibility to ARTs-related liver toxicity [23]. Therefore, prior to the administration of ART, patients should be tested for the presence of these infections. The aim for this study is to investigate the prevalence of HCV and/or HBV coinfection in Iranian HIV-infected individuals.

## Materials & methods

### Study patients

From September 2015 to June 2018, 198 consecutive Iranian HIV-positive individuals who were referred to hospitals affiliated with Iran University of Medical Sciences (IUMS), Tehran, Iran, were entered to this study. The research was approved by the IUMS’ Ethical Committee, and all of the studied population were informed about this survey, and a written informed consent was obtained from all the subjects and also from parents of HIV-infected children in this cross-sectional study.

### Collection of the specimens

5 ml of the patient’s blood was taken from each participant into an EDTA-containing vacutainer tube. After separation of the plasma by centrifugation (5 min at 3000 RPM), plasma was stored at -80°C until analysis. Plasma specimens from ten individuals who were infected with HCV, and ten subjects who were infected with HBV were used as positive controls, and also plasma samples from ten healthy blood donors were used as negative controls for the experiments.

### Serologic tests by enzyme immunoassay

Serological markers of HBV and HCV infection such as HBsAg, HBeAg, hepatitis B e-antibody (HBeAb), hepatitis B core antibody (HBcAb) and anti-hepatitis C virus antibodies (anti-HCV) Abs were tested by the commercial enzyme immunoassay kits (DIA. PRO, Milano, Italy), according to the manufacturer’s protocols.

### HBV viral load & HBV genotyping

HBV viral load was assessed in 500 μl of the studied subjects plasma specimens using the high pure DNA extraction kit and COBAS TaqMan 48 kit (Roche Diagnostics, CA, USA) according to the manufacturer’s procedure [24]. This test is a real-time PCR assay that is based on dual-labeled hybridization probe that targets two regions (precore and core) of HBV. The detection limit of the COBAS TaqMan 48 kit is 6 to >1 × 10^8^ IU/ml [25]. The HBV genotyping was determined in HBV-DNA-positive specimens using the INNO-LiPA™ HBV kit (Innogenetics, Ghent, Belgium) according to the manufacturer’s protocols [26].

### HCV detection by reverse transcriptase-nested PCR method & HCV genotyping with restriction fragment length polymorphism assay

To detect genomic HCV-RNA in the plasma samples of studied subjects, the viral RNA was isolated from 140 μl of plasma using the QIAamp Viral RNA isolation kit (Qiagen GmbH, Hilden, Germany) based on the manufacturer’s procedure. The quantity and quality of the extracted RNA was evaluated using the NanoDrop™ spectrophotometer (Thermo Fisher Scientific, Wilmington, NC, USA) [27]. The HCV-RNA was detected in extracted RNA of plasma samples by the reverse transcriptase-nested PCR (RT-nested PCR) assay using two sets of primers for the 5′-non-translated region (5′-NTR) of HCV, as previously described in detail [28,29]. The amplified PCR products of subjects’ samples, negative and positive control specimens, and 100 bp DNA size marker were electrophoresed on a 2.2% gel agarose and then stained with syber green and visualized by a UV transilluminator.

The genotyping of HCV was determined in HCV-positive samples with restriction fragment length polymorphism (RFLP) assay, based on a protocol that previously described in detail [28,29].

### Statistical analysis

The statistical analysis was performed using SPSS software version 20 (SPSS Inc., IL, USA). The Kolmogorov–Smirnov test was conducted to determine the quantitative variables’ normality. The analysis of continuous variables was done using Kruskal–Wallis and one-way analysis of variance (ANOVA) tests. The statistical differences between the two groups were evaluated by Fisher’s exact test and Chi-square test when appropriate. p-values <0.05 were considered statistically significant.

## Results

From September 2015 to June 2018, a total of 198 HIV-infected individuals (anti-HIV Abs and HIV-RNA positive) were enrolled in this cross-sectional study. The mean age of subjects was 35.3 ± 13.5 years (a range of 1–68 years old). Out of 198 studied individuals, 126 (63.6%) were male. Complete information of the demographic, laboratory and epidemiological characteristics were presented in Table 1.

**Table 1.** Demographic and laboratory parameters of the studied subjects with HIV infection.

Parameters	Male	Female	Total	p-value
No. of patients	126 (63.6%)	72 (36.4%)	198 (100%)	
Age (year) ± SD	36.2 ± 12.3 (2–69)	33.6 ± 15.4 (1–62)	35.3 ± 13.5 (1–68)	0.188 t test
Laboratory parameters:				
CD4 count	471.5 ± 595.0 (25–4463)	452.7 ± 231.8 (69–1028)	464.9 ± 497.1 (25–4463)	0.077 Mann–Whitney U
HIV viral load (IU/ml) (median)	558.0 (43–2,055,673)	2142.5 (67–129,422)	620.0 (43–2,055,673)	0.251 Mann–Whitney U
ALT (IU/l)	53.3 ± 22.9 (17–178)	40.4 ± 16.3 (9–76)	48.6 ± 21.6 (9–178)	<0.001^†^ Mann–Whitney U
AST (IU/l)	48.9 ± 21.3 (8–169)	37.0 ± 15.7 (8–66)	44.4 ± 20.2 (10–169)	<0.001^†^ Mann–Whitney U
HCV-RNA	39 (31.9%)	3 (4.2%)	42 (21.2%)	<0.001^†^ Fisher’s Exact test
Anti-HCV Ab	79 (62.7%)	6 (8.3%)	85 (42.9%)	<0.001^†^ Fisher’s Exact test
HBV DNA	8 (6.3%)	0 (0.0%)	8 (4.0%)	0.053^†^ Fisher’s Exact test
HBsAg	18 (14.3%)	7 (9.7%)	25 (12.6%)	0.385 Fisher’s Exact test
Anti-HBcAb	50 (39.7%)	5 (6.9%)	55 (27.8%)	<0.001^†^ Fisher’s Exact test
HBeAg	4 (3.2%)	0 (0.0%)	4 (2.0%)	0.299 Fisher’s Exact test
Anti-HBeAb	9 (7.1%)	1 (1.4%)	10 (5.1%)	0.097 Fisher’s Exact test
Epidemiological parameters:				
History of having unprotected sex	59 (46.8%)	16 (22.2%)	75 (37.9%)	0.001^†^ Fisher’s Exact test
History of imprisonment	72 (57.1%)	0 (0.0%)	72 (36.4%)	<0.001^†^ Fisher’s Exact test
IDUs	79 (62.7%)	2 (2.8%)	81 (40.9%)	<0.001^†^ Fisher’s Exact test
IDU sexual partner	0 (0.0%)	46 (63.9%)	46 (23.2%)	<0.001^†^ Fisher’s Exact test
History of transfusion	15 (11.9%)	2 (2.8%)	17 (8.6%)	0.034^†^ Fisher’s Exact test
History of tattooing	39 (31.0%)	3 (4.2%)	42 (21.2%)	<0.001^†^ Fisher’s Exact test
History of needle stick	33 (26.2%)	1 (1.4%)	34 (17.2%)	<0.001^†^ Fisher’s Exact test
Mother-to-child infection	10 (7.9%)	8 (11.1%)	18 (9.1%)	0.453 Fisher’s Exact test

† Statistically significant.

Ab: Antibody; ALT: Alanine aminotransferase; Anti-HBcAB: Anti-hepatitis B core antibody; Anti-HBeAb: Hepatitis B e-antibody; AST: Aspartate aminotransferase; CD4 count: CD4+T cell count; HBeAg: Hepatitis B e-antigen; HBsAg: Hepatitis B virus surface antigen; IDU: Injection drug user; SD: Standard deviation.

A significant association was observed between the sex of the participants and alanine aminotransferase (ALT), aspartate aminotransferase (AST) level, anti-HCV Abs, HCV-RNA (p < 0.001) in plasma samples, and also in epidemiological parameters such as history of having unprotected sex, history of imprisonment, injection drug users (IDUs), IDU sexual partners, history of tattooing, history of needle stick (p < 0.001) and history of transfusion (p = 0.034) (Table 1). A significant relationship was observed between coinfection with HCV or HBV in HIV-infected patients and CD4^+^ T-cell count (p = 0.044), in other words, CD4^+^ T-cell count was very low in patients with these coinfections. A strong association was observed between the sex of the participants and level of education (p = 0.042) and marital status (p < 0.001) (Table 2).

**Table 2.** Level of education marital status of the studied injection drug users with HIV infection.

Parameters	Male	Female	Total	p-value
No. of patients	126 (63.6%)	72 (36.4%)	198 (100%)	
Level of education				
Under diploma	74 (58.7%)	54 (75.0%)	128 (64.6%)	0.042^†^ Chi-square test
Diploma	37 (29.4%)	13 (18.1%)	50 (25.3%)	
Bachelor	8 (6.3%)	5 (6.9%)	13 (6.6%)	
Master and doctorate	7 (5.6%)	0 (0.0%)	7 (3.5%)	
Marital status:				
Single	67 (53.2%)	16 (22.2%)	83 (41.9%)	<0.001^†^ Chi-square test
Married	48 (38.1%)	37 (51.4%)	85 (42.9%)	
Divorced	11 (8.7%)	7 (9.7%)	18 (9.1%)	
Widow	0 (0.0%)	12 (16.7%)	12 (6.1%)	

† Statistically significant.

85 (42.9%) of studied subjects were positive for anti-HCV Abs in plasma samples; and 42 (21.2%) had detectable HCV-RNA in the plasma (Table 1). The HCV genotyping was performed using RFLP assay for HCV-RNA-positive specimens, and the results of HCV genotyping are presented in Table 3.

**Table 3.** Results of HCV genotyping of the studied subjects with HIV/HCV coinfection.

HCV genotypes	Male (n)	Male (%)	Female (n)	Female (%)	Total (n)	Total (%)
1a	17	43.6%	-	-	17	40.5
1b	6	15.4%	1	33.3%	7	16.7
3a	12	30.8%	2	66.7%	14	33.3
1a/3a	2	5.1%	-	-	2	4.8
1b/3a	2	5.1%	-	-	2	4.8
Total	39	100%	3	100%	42	100%

n: Number.

Eight (4.0%) of the studied cases had detectable HBV-DNA in the plasma samples (Table 1). The HBV genotyping was carried out for these samples by the INNO-LiPA HBV kit. All these patients were infected by HBV genotype D. 55 (27.8%) of the participants were HBcAb positive, and a strong relationship was observed between the sex of the studied participants and anti-HBcAb in plasma specimens (p < 0.001) (Table 1).

This survey demonstrated that none of the Iranian HIV-infected individuals were HIV-RNA/HCV-RNA/HBV-DNA positive simultaneously, 21.2% were HIV-RNA/HCV-RNA positive and 4.0% were HIV-RNA/HBV-DNA positive.

No significant association was observed between the HIV viral load in coinfected patients and monoinfected patients (p = 0.054). Nine (4.4%) of the studied HIV-infected patients were HBsAg and anti-HCV Ab positive. All the information about demographic and laboratory parameters of these patients are presented in Table 4.

**Table 4.** All the information about demographic and laboratory parameters of the patients with HIV/HCV or HIV/HBV coinfection.

Patients	Sex/Age	HIV viral load copies/ml	CD4 count	ALT^1^	AST^2^	HBsAg	HBcAb	HBeAg	HBeAb	HBV-DNA	HCV Ab	HCV-RNA	HCV-genotype
P- 48	F/31	71	271	81	73	+	−	−	−	+	+	−	−
P- 112	M/35	4965	118	44	48	+	+	+	−	+	+	−	−
P- 113	M/35	80	211	53	59	+	+	−	−	−	+	+	3a
P- 117	M/48	60	365	89	91	+	+	−	−	−	+	+	1a
P- 133	M/45	7819	141	30	32	+	+	−	−	−	+	−	−
P- 136	M/32	667	125	80	83	+	+	−	−	−	+	−	−
P- 188	M/31	4700	132	42	47	+	+	+	−	+	+	−	−
P- 195	M/49	8175	122	37	39	+	+	−	−	−	+	−	−
P- 196	M/28	890	144	64	68	+	+	−	−	−	+	−	−

Ab: Antibody; ALT: Alanine aminotransferase; AST: Aspartate aminotransferase; CD4 count: CD4+T cell count; HBcAb: Hepatitis B core antibody; HBeAb: Hepatitis B e-antibody; HBeAg: Hepatitis B e-antigen; HBsAg: Hepatitis B virus surface antigen.

## Discussion

Despite the existence of successful prevention and treatment methods, the simultaneous infection of HIV, HBV and HCV is still a worldwide health issue. With the use of antiretroviral medicines and longer life expectancy in HIV-infected patients, the complications of this chronic disease and its intersection with other viral infections are more evident [30]. In Iran, the prevalence of HIV and other blood-related viral infections, such as HCV is relatively low in the general population [31]. The present survey was conducted on 198 individuals who were infected with HIV to investigate the molecular epidemiology of HCV/HBV coinfection in these individuals. This study showed that none of the HIV-infected people were HIV-RNA/HCV-RNA/HBV-DNA positive simultaneously, 4.0% were HIV-RNA/HBV-DNA positive and 21.2% were HIV-RNA/HCV-RNA positive.

HCV infection is more common in people infected with HIV than in HIV-negative individuals [32]. The rate of HCV/HIV coinfection is different around the world and is heavily dependent on geographical location, socioeconomic conditions of that particular location and high-risk groups [32]. Nearly 37 million people are infected with HIV so far and about 70 million people all over the world are infected with HCV [2,13]. Approximately 2,278,400 people have HIV/HCV coinfection in the world, and about 62% of them are people that have been infected through injecting drugs [32]. The present study showed that approximately 81 (40.9%) of the patients are those who have injected drugs and about 46 (23.2%) of them are those who had IDU sexual partner (that includes only women) (Table 1). The epidemiologic parameters such as injection drug abuse, needle sharing, tattooing, history of imprisonment and history of having unprotected sex revealed higher prevalence of HCV-coinfection in these patients compared with monoinfected cases. In the coinfected patients, the level of liver enzymes ALT/AST was significantly high. While the first way of HIV transmission in Iran is from sharing injection needles among IDUs [33], HIV transmission cases in IDUs has begun to decline from 2005, a trend that has continued so far, and today HIV transmission through unprotected sexual contact is increasing (36.8%) [34,35]. In this study the number of IDUs was 81 (40.9%). Since the transmission of HCV by sexual contact is a rare phenomenon, the number of people coinfected with HCV/HIV is expected to decrease in the future [3,36]. In the current study, 75 (37.9%) of the HIV-positive patients had a history of unprotected sex and probably the HIV virus in these patients transferred through unprotected sex. Perhaps this is the reason for the decrease in the number of patients with HCV/HIV coinfection (21.2%) compared with previous reports [37,38]. Of course, in recent years, the increase in the transmission of HCV in males that have sexual intercourse with other males has been seen due to high-risk sexual behaviors. In addition, there have been cases of HCV spontaneous clearance in people who inject drugs (PWID) after ART [39,40].

The HCV genotyping on the plasma specimens showed a prevalence for subtypes, 1a (40.5%), 1b (16.7%) and 3a (33.3%). In four samples, the divergence genotype detected a mixed infection of two subtypes (1a/3a–1ab/3a) (Table 3). HCV genotyping had been performed on serum samples of patients coinfected with HIV/HCV in different countries, for example, in the Chinese (1b [27.2%], mixed [23.7%], 6a [15.8%], 3b [12.3%], 1a [8.8%], 3a [4.4%], 2a [4.4%] and 2b [3.5%]); in Indians (1b, 1a, 3a and 3b [51.1%], [17.7%], [22.2%] and [8.8%]); in Brazilians (1a, 1b and 3a [47.2%], [11.1%] and [41.7%], respectively); and in Iranians (1a, 3a and 3b [75.9%], [18.5%] and [5.6%], respectively) [41–43]. According to various reports from around the world, it seems that more research is required in this field with a wider population.

Like HCV infection, all HIV-infected patients should be screened for HBV infection. Cirrhosis, HCC and hepatotoxicity after ARTs are the effects of HBV/HIV coinfection [44]. HBV vaccination should be done in all HIV individuals with HBV-negative laboratory tests. Similar to HCV, HCC occurs in HBV/HIV-coinfected patients without cirrhosis [45]. HBV viral load is higher in HIV/HBV-coinfected patients than HBV-monoinfected individuals [46]. The present study found that 55 (27.8%) of these patients have anti-HBcAb and 25 (12.6%) of them have HBsAg. In some people, antibodies of the HBV core can be detected without anti-HBsAg and HBeAg [47]. Our study confirms previous reports that male subjects are at high risk of developing HBV infection. Typical methods for the transmission of both HBV and HIV are the sexual pathway and injection drug abuse [48], while transmission of HCV by sexual pathway is unusual and given that in recent years the pathway for HIV transmission has changed, the prevalence of HCV is changing, but a significant change in the prevalence of HBV is unlikely to occur [49]. In a study on blood donors with an NAT test in Tehran, the incidence and residual risk for HIV was lower than those in developed countries, whereas HBV and HCV was higher compared to developed countries. In the Iranian population, HIV infection is lower than the other countries, and screening tests are effective for blood donors. In the case of HCV, an increased incidence of HCV infection has been observed in the Iranian society and in blood donors in recent years; this may be due to the highest number of IDUs in Iran compared to other Middle Eastern countries [50]. Incidence and high residual risk in Iran indicates the nature of the endemic HBV virus. Due to the launch of HBV vaccination in Iran in 1993, we have to wait for the effects of the vaccination among the Iranian population. Based on that study, blood donors should have a more accurate technique similar to the accurate NAT-screening techniques [51]. In patients with coinfection of HIV with HCV and/or chronic HBV, progressive liver fibrosis, cirrhosis and HCC can occur and coinfection of HIV with HBV and/or HCV can affect the management of HIV infection and complicate it [44,52]. Therefore, it is best to identify infection of hepatitis as quickly as possible.

The result of this study revealed that none of the participants were HIV-RNA/HCV-RNA/HBV-DNA positive simultaneously. To the best of our knowledge, the current survey is the first research that has analyzed the presence of the molecular epidemiology of HCV/HBV coinfection in Iranian HIV-infected individuals; therefore, the results of this study cannot be compared with the result of other Iranian research. There have been reports of coinfection with HBV and HCV in HIV-positive people, for example, 0.5% in Singapore [53], 0.62% in Germany [54] and 1.7% in Serbia [55]. It seems that further research focusing on this issue is needed.

Although there are studies that indicate seroprevalence of HIV/HCV/HBV coinfection in Iran. For example, Bakhti *et al.* found that 8% of HIV-infected individuals are coinfected with HCV/HBV (HBsAg and anti-HCV Ab positive), and in a meta-analysis, Bagheri Amiri *et al.* reported that coinfection of HIV/HBV/HIV was close to zero in the general population, street children and healthcare workers, while it peaked to 1.25% in PWID [56]. This study revealed that in Iranian HIV-infected individuals, 4.4% of the individuals are seropositive for HBV/HCV (HBsAg and anti-HCV Ab positive).

According to a previous study, prevalence of cirrhosis in HIV/HBV/HCV triple-infected patients was higher than HIV/HBV- or HIV/HCV-coinfected individuals [57]. Therefore, prevention programs for HIV/HBV/HCV coinfection are in need of development.

## Conclusion

This study reveals that there is a high prevalence of HCV infection (21.2%) in HIV-infected individuals (HIV-RNA/HCV-RNA positive), as well as 4% of these people infected with HBV (HIV-RNA/HBV-DNA positive). Also, the result of this survey highlighted that none of the HIV-infected subjects were HCV-RNA/HBV-DNA positive simultaneously. Therefore, it seems that in HIV-positive patients, in addition to routine diagnosis of various infection diseases, HCV and HBV infection should be considered as well. This is because infection with these viruses (HCV and HBV) in HIV-infected patients may cause complex problems and postpone the global eradication of HIV infection.

**Summary points**

- The present study clarified that none of the Iranian HIV-infected individuals were HIV-RNA/HCV-RNA/HBV-DNA positive simultaneously.
- 21.2% of the Iranian HIV-infected individuals were HIV-RNA/HCV-RNA positive, and 4.0% were HIV-RNA/HBV-DNA positive.
- It seems that designing a research focusing on the diagnosis of HBV and HCV infections in HIV-infected individuals can be valuable.
